# Adaptation and validation of the Cyprus version of the Practice Environment Scale of the Nursing Work Index: a methodological study

**DOI:** 10.1186/s13104-018-3896-2

**Published:** 2018-11-03

**Authors:** Georgios Efstathiou, Christos Andreou, Haritini Tsangari, Maria Dimitriadou, Evridiki Papastavrou

**Affiliations:** 1 Educational Sector, Nursing Services, Ministry of Health, Nicosia, Cyprus; 2 Department of Nursing, Cyprus University of Technology, Limassol, Cyprus; 3 University of Nicosia, Nicosia, Cyprus

**Keywords:** Adaptation, Cyprus, Nurses, RN4CAST, Validation, Working environment

## Abstract

**Objective:**

The purpose of this study was to adapt and validate the Revised Practice Environment Scale of the Nursing Work Index, to be utilized among the Cyprus nurses’ population. Validated research scales are valuable tools in the hands of researchers in their attempt to recommend alterations within health care systems, especially during reform periods.

**Results:**

Internal consistency reliability of the scale was satisfactory (alpha = 0.94). Exploratory factor analysis produced a five-factor structure solution. Experts in the field provided further guidance. The findings demonstrated that the Cyprus version of the Revised Practice Environment Scale of the Nursing Work Index is a reliable and valid research instrument, however differences with previous studies on the factor structure were observed, mainly due to cultural reasons. As a valid and reliable scale, it can capture the views of nurses concerning their work environment. The Cyprus version of the Revised Practice Environment Scale of the Nursing Work Index can be used by nurse managers and policy makers to introduce changes that may improve the quality of nursing care provided and enhance the patients’ satisfaction from the health system as a whole.

## Introduction

Changes in health care systems result in the necessity to focus on forecasting models for nursing workforce reforms. Evidence indicates that current health workforce planning methods have poor results and do not enable health care managers to predict needs regarding the nursing profession. They also fail to allow policy interventions to be implemented satisfactorily to avoid staffing shortages [1–3]. The European Registered Nurse Forecasting (RN4CAST) research project was launched in 2009 aiming to investigate the nursing working environment, the nursing staff competency, workload, burnout and job satisfaction as well as the associations of those nursing variables towards patients’ outcomes. The present Cyprus RN4CAST survey was a replication of the initial protocol. Data collection regarding nurses’ perceptions on their working environment was accomplished by using the 32-item, 5-factor structure Revised Practice Environment Scale of the Nursing Work Index (PES-NWI-R). The PES-NWI-R appears cross-culturally applicable, it enables the measurement of differences between hospital organizations and it has a clear association with health care outcomes [4–7]. The multi-lingual use of the PES-NWI-R among nurses of different cultural settings led to several methodological studies [5, 8].

Despite the wide use of PES-NWI-R, there is a scarcity of publications reporting modifications of the instrument necessary for use in different settings and countries [5, 9]. Acknowledging that the establishment of cross-culturally relevant, valid, reliable and rigorously adapted instruments for international research is crucial for the quality of research findings, the aim of this study was to adapt and validate the PES-NWI-R for use among Cypriot nurses.

## Main text

### Methods

This is a methodological study, conducted to adapt and validate the PES-NWI-R [3, 4, 6, 10], specifically the Greek version. Although the Greek language is spoken in Cyprus, differences exist on the use of medical and nursing terminology. Therefore, adaptation of the Greek version was necessary.

### Cross-cultural adaptation

#### Face and content validity

The Greek version of the PES-NWI-R was examined. Words that could confuse Cypriot nurses due to the use of different terminology in daily practice, were replaced (Table 1). Every effort was made to achieve semantic and conceptual equivalence to the Greek version. This first draft was evaluated by six clinical nurses; some suggestions for modifications were adopted.

**Table 1:** Examples of word/phrases changes

Original version	Greek version vs Cyprus version
Items 6, 9, 25, 29—*registered* nurses	A different word to describe *registered* was deemed necessary (Greece version *πιστοποιημένοι* {*pistopioimeni*}, Cyprus version *εγγεγραμμένοι* {*eggegrameni*}) to allow safe understanding of the concept
Item 10—*nurse manager*	Different words to describe *nurse manager* was deemed necessary (Greece version διοικητής νοσηλευτικής υπηρεσίας {dioikitis nosileftikis ipiresias}, Cyprus version προϊστάμενος/η νοσηλευτικός/η λειτουργός {proistamenos nosileftikos litourgos}) to allow safe understanding of the concept
Item 20—clinically *competent*	Different word to describe competent was deemed necessary (Greece version ικανοί {ikani}, Cyprus version επαρκείς {eparkis})
Item 22—*nurse manager*	Different words to describe *nurse manager* was deemed necessary (Greece version διοικητής μονάδας {dioikitis monadas}, Cyprus version προϊστάμενος τμήματος {proistamenos tmimatos})

Changes are presented in the Greek language (Greek and Cypriot version as well as how it is pronounced in English)

Content Validity Index (CVI) was implemented for assessment of the scale’s content validity. Ten experts who were nurses with postgraduate qualifications, evaluated the preliminary Cypriot version in terms of item importance and relevance, using a 4-point Likert scale: 1 = *not relevant*, 2 = *somewhat relevant*, 3 = *quite relevant*, 4 = *highly relevant.* CVI was satisfactory (CVI = 0.97).

### Pilot study

The instrument was piloted among a convenience sample of 95 clinical nurses. Only minor changes were required at this stage. Internal consistency for the whole scale was alpha = 0.93 and for subscales was alpha = 0.71–0.88.

### Field study

To further test the instrument, it was distributed to 950 nurses working on medical and surgical wards; distribution took place during March and May 2015. Eligibility criteria were: willingness to participate as well as at least 1 year of professional experience. Research associates distributed the questionnaires and provided information on the scope of the study. Response rate was 65% (N = 507). The Statistical Package for the Social Sciences (SPSS) version 20 was used for analysis. Cronbach’s alpha was used to test the scale’s internal consistency and exploratory factor analysis (EFA) with Varimax rotation was used to break the list of 32 statements into groups of related variables and test the scale’s construct validity. The Kaiser–Meyer–Olkin (KMO) Measure of Sampling Adequacy was 0.949, while Bartlett’s test of Sphericity had a p-value < 0.001, indicating that the data were appropriate for a factor analysis.

### Ethical considerations

The protocol of the study was reviewed by the Cyprus National Bioethics Committee (EEBK EΠ 2014.01.3); the Ministry of Health authorized the distribution of the questionnaires in all public hospitals. Nurses participated voluntarily, maintained the right to withdraw and verbally acknowledged that completion of a questionnaire would be considered as informed consent. Anonymity and confidentiality were preserved. Data were kept safely. Permission to replicate the study was obtained by Professor Sermeus (personal contact).

### Results

#### Participants’ characteristics

Most of the participants were young (64% between the ages of 25–34), female (67%), trained in Cyprus (93%) and holding a University Degree in Nursing (98%). Most were working on a full-time basis (98%) with a mean work experience of 11.4 years (SD 10.1).

#### Internal consistency and EFA

Internal consistency was satisfactory (alpha = 0.94). The EFA extracted five factors (all eigenvalues > 1), explaining a total of 56% of the variation. All item loadings in their respective factor were over 0.3 (Table 2). Every item was relevant to the other items included in the corresponding factor, therefore—for the benefit of the study—all items were retained.

**Table 2:** Items loadings in corresponding factors (N = 507)

	F1	F2	F3	F4	F5
1. Adequate support services allow me to spend time with my patients	0.655				
2. Doctors and nurses have good working relationships		0.614			
3. A supervisory staff that is supportive of nurses				0.784	
4. Active staff development or continuing education programs for nurses				0.555	
5. Career development/clinical ladder opportunity				0.370	
6. Opportunity for registered nurses to participate in policy decisions				0.369	
7. Doctors value nurses’ observations and judgments		0.734			
8. Enough time and opportunity to discuss patient care with other nurses	0.706				
9. Enough registered nurses on staff to provide quality patient care	0.700				
10. A nurse manager who is a good manager and leader				0.648	
11. A chief nursing officer who is highly visible and accessible to staff				0.402	
12. Enough staff to get the work done	0.738				
13. Doctors recognize nurses’ contributions to patient care		0.729			
14. Praise and recognition for a job well done				0.327	
15. High standards of nursing care are expected by the management					0.638
16. A chief nursing officer is equal in power and authority to other top level hospital executives					0.413
17. A lot of team work between nurses and doctors		0.597			
18. Opportunities for advancement			0.413		
19. A clear philosophy of nursing that pervades patient care environment					0.448
20. Working with nurses who are clinically competent					0.617
21. Doctors respect nurses as professionals		0.746			
22. A nurse manager who backs up the nursing staff in decision making, even if the conflict is with a doctor				0.453	
23. Management that listens and responds to employee concerns	0.414				
24. An active quality assurance program			0.394		
25. Registered nurses are involved in the internal governance of the hospital (e.g. practice and policy committees)			0.420		
26. Collaboration between nurses and doctors		0.754			
27. A preceptor program for newly hired nurses			0.653		
28. Nursing care is based on a nursing rather than a medical model			0.595		
29. RNs have the opportunity to serve on hospital and nursing committees			0.575		
30. Doctors hold nurses in high esteem		0.694			
31. Written, up-to-date care plans for all patients			0.575		
32. Patient care assignments that foster continuity of care (i.e. the same nurse care for the same patient from 1 day to the next)	0.485				

Factors were named according to the items included within each factor (Table 3). Further testing by two experienced registered nurses, validated the above item distribution.

**Table 3:** Labeling of factors and corresponding items

Factor 1, Staffing and resource adequacy
Adequate support services allow me to spend time with my patients
Enough time and opportunity to discuss patient care with other nurses
Enough registered nurses on staff to provide quality patient care
Enough staff to get the work done
Management that listens and responds to employee concerns
Patient care assignments that foster continuity of care (i.e. the same nurse care for the same patient from 1 day to the next)
Factor 2, Nurse–doctor relations
Doctors and nurses have good working relationships
Doctors value nurses’ observations and judgments
Doctors recognize nurses’ contributions to patient care
A lot of team work between nurses and doctors
Doctors respect nurses as professionals
Collaboration between nurses and doctors
Doctors hold nurses in high esteem
Factor 3, Nursing policy
Opportunities for advancement
An active quality assurance program
Registered nurses are involved in the internal governance of the hospital (e.g. practice and policy committees)
Nursing care is based on a nursing rather than a medical model
RNs have the opportunity to serve on hospital and nursing committees
Written, up-to-date care plans for all patients
A preceptor program for newly hired nurses
Factor 4, Nursing management and development
A supervisory staff that is supportive of nurses
Active staff development or continuing education programs for nurses
Career development/clinical ladder opportunity
Opportunity for registered nurses to participate in policy decisions
A nurse manager who is a good manager and leader
A chief nursing officer who is highly visible and accessible to staff
Praise and recognition for a job well done
A nurse manager who backs up the nursing staff in decision making, even if the conflict is with a doctor
Factor 5, Nursing competency
High standards of nursing care are expected by the management
A chief nursing officer is equal in power and authority to other top level hospital executives
A clear philosophy of nursing that pervades patient care environment
Working with nurses who are clinically competent

### Discussion

The five-factor structure Cyprus version of PES-NWI-R has acceptable CVI and internal consistency (alpha = 0.94). All items of the original version have been retained. Although factorial structure is similar to the original 5 factor-structure of the PES-NWI-R [10, 11], the items within the factors are different. Similar differences in item structure level have been observed in previous studies [12]. Such differences can be attributed to cultural differences among countries, as well as to varying health care and management systems. This is an important consideration, as Cyprus has been, during the current study, in a transitional period concerning its health care system [13]. This might have influenced the nurses’ responses because many changes and political decisions as well as conflicts between unions and government have taken place. Nurse researchers are challenged to further investigate in a more global perspective the concept of nursing working environment, since, once again, it has been shown that is considered differently among cultures. Current situations and local condition seem to play a vital role on how the nursing environment is described, a fact that policy makers need to be aware of, if they want to achieve an acceptable nursing working environment.

In this study, factor one consists of six items, relevant to *Staffing and Resources Adequacy.* This is in line with Lake’s study [10], Klopper et al. [6] and Parker et al. [14] studies except item named *Working with nurses who are clinically competent* which, in the present study, was loaded within a different factor, namely Nursing Competency. The move of this item to another factor can be attributed to the current situation in Cyprus; almost all nurses are holders of at least Bachelor’s in nursing. Competency is seen by nurses in this study as having to do high level of standards or being able to have equal power with other top-level hospital executives, which is explained by the position of high academic qualifications.

Factor two of the present study includes seven items, dealing on nurse–doctor relationship. It includes items on how doctors and nurses cooperate but also on what doctors believe—from the nurses’ perspective—about nurses. This factor is the only one that corresponds in its entity with the findings by Klopper et al. [6] and the same items (but not all) are included in the study by Parker et al. [14], Lake [10] and Van Bogaert et al. [15], whereas in Chiang and Lin [12] study, all items were scattered in various factors, probably due to cultural differences and perceptions in the Chinese population of nurses. The relationship between nurses and doctors can, probably, be affected by historical reasons and how both professions developed (together, in parallel etc.) but also on how the society in general perceives the roles and relationship between the two professions [16]. In some cultures, the doctor’s opinion is decisive and under this status, little opportunity is given to nurses to work as autonomous health professionals [17, 18]. In the Cyprus context, many nurses hold up to Ph.D. qualifications, allowing them to act more autonomously, influencing in this way their perceptions regarding their working environment.

Factor three in this study is named *Nursing Policy* as it includes items that do not correspond with previous labelling of the factors in other studies. For example, the items included in this factor were included in Lake’s study [10] into different factors (Nurse Participation in Hospital affairs and Nursing Foundations for quality of care). However, in this study, these items were grouped in one factor, implying that in the Cyprus environment they form a different idea. They look on how the nursing profession is ruled, what policies exist and how nurses are involved in internal governance within the hospital. Thus, this factor, was named *Nursing Policy* since it reflects the general policy in nursing that covers the profession. It should be noted that in 2009, a directorate of Nursing Services was set up and is working at Ministerial level. One of its missions, is to develop policies, protocols and clinical guidelines that assist nurses to perform their duties. The existence of the directorate, which is independent from other directorates, has facilitated the perception of nurses in Cyprus as autonomous health professionals, that develop their policy and regulate the profession.

Factor four of the present study includes items that deal with management and professional development. It was also labelled differently from previous studies since its content is different, as items from the present study were scattered in different factors in previous studies (e.g. in Parker’s et al. study [14] items *a nurse manager who backs up the nursing staff in decision making, even if conflict is with a doctor* and *Active staff development or continuing education programme for nurses*). Once again it is obvious that the effect the possession of high qualifications among nurses in Cyprus has on their perception regarding the importance of professional development of nurses. This has led to grouping of all relevant items regarding this topic under one factor.

Factor five includes four items, all relevant to nursing quality and what is expected from nurses to be competent/be considered competent e.g. *working with nurses who are clinically competent.* This is a totally new factor, since in previous studies these items were included in different factors. In Parkers et al. study [14], quality was also seen from the perspective of the existence of a nursing model on which nursing care is based on, whereas Klopper et al. [6] suggested that quality also included a clear philosophy of nursing and the existence of a preceptor programme for new nurses. These findings reflect the differences between health care systems with reference to what is expected to be included in quality nursing care and how this is delivered and presented. For example, in the perception of Cypriot nurses, being competent to practice nursing is not relevant to staffing and resources but is grouped with other concepts like high standards and equal authority with other professionals. This is an important consideration, especially in current worldwide trend of practicing nursing in other countries. Nurses originating from other parts of the world and different health care and educational systems might find challenging the effort to become part of a new system.

### Conclusions

This study showed that the Cyprus version of PES-NWI-R has satisfactory psychometric properties. Reliability findings are adequate, and a new factor structure is proposed. This structure is solid, since all factors include more than three items, they have adequate loading to their respective factor and have semantic relationship. The Cyprus version can be used for further testing the nurses working environment in Cyprus in order to facilitate changes but also to persuade policy-makers about the importance of nurses as part of the health system. Future studies may also focus into an in more in-depth testing of the PES-NWI-R, by replicating our study, to confirm its factor structure.

## Limitations

To follow the RN4CAST protocol, this study was confined to nurses working in surgical-medical departments. Therefore, further research in other clinical settings is suggested. A convenience sample was used, limiting the generatability of findings.

## Abbreviations

RN4CAST: Nurse Forecasting in Europe
PES-NWI-R: Revised Practice Environment Scale of the Nursing Work Index
ICN: International Council of Nurses
CVI: Content Validity Index
